# TGM2-Mediated Autophagy Contributes to the Radio-Resistance of Non-Small Cell Lung Cancer Stem-like Cells

**DOI:** 10.3390/biomedicines12102231

**Published:** 2024-09-30

**Authors:** Qian Wang, Qiuning Zhang, Xiaohu Wang, Hongtao Luo, Tianqi Du, Luyao Wu, Mingyu Tan, Yanliang Chen, Xun Wu, Shilong Sun, Zhiqiang Liu, Yi Xie, Wenzhen Yuan

**Affiliations:** 1 The First School of Clinical Medicine, Lanzhou University, Lanzhou 730030, China; qianwang21@lzu.edu.cn (Q.W.);; 2 Institute of Modern Physics, Chinese Academy of Sciences, Lanzhou 730030, China; 3 Graduate School of the Chinese Academy of Sciences, Beijing 101499, China

**Keywords:** non-small cell lung cancer, cancer stem-like cells, CD44, radiation sensitivity, transglutaminase 2, autophagy

## Abstract

**Objectives**: Cancer cells with ‘stemness’ are generally resistant to chemoradiotherapy. This study aims to compare the differences in radiation sensitivity of A549 and CD44^+^A549 stem-like cells to X-rays and carbon ion radiation (C-ions), and to find a target that can kill cancer stem-like cells (CSCs) of non-small cell lung cancer (NSCLC). **Methods**: The study used two cell lines (A549 and CD44^+^A549). The tumorigenicity of cells was tested with animal experiments. The cells were irradiated with X-rays and C-ions. Cell viability was detected using the CCK-8 and EdU assay. A liquid chromatograph-mass spectrometer (LC–MS) helped detect metabolic differences. Protein and mRNA expression were detected using a Western blot, reverse transcription-quantitative (RT-qPCR), and PCR array. The autophagic activity was monitored with a CYTO-ID^®^ Autophagy Detection Kit 2.0. Immunofluorescence and co-immunoprecipitation helped to observe the localization and interaction relationships. **Results:** First, we verified the radio-resistance of CD44^+^A549 stem-like cells. LC-MS indicated the difference in autophagy between the two cells, followed by establishing a correlation between the radio-resistance and autophagy. Subsequently, the PCR array proved that TGM2 is significantly upregulated in CD44^+^A549 stem-like cells. Moreover, the TGM2 knockdown by small interfering RNA could decrease the radio-resistance of CD44^+^A549 cells. Bioinformatic analyses and experiments showed that TGM2 is correlated with the expression of CD44 and LC3B. Additionally, TGM2 could directly interact with LC3B. **Conclusions:** We established the CD44-TGM2-LC3 axis: CD44 mediates radio-resistance of CD44^+^A549 stem-like cells through TGM2 regulation of autophagy. Our study may provide new biomarkers and strategies to alleviate the radio-resistance of CSCs in NSCLC.

## 1. Introduction

Lung cancer has the highest morbidity and mortality rate in the world. Non-small cell lung cancer (NSCLC) accounts for 80% to 85% of all lung cancer patients, including adenocarcinoma, squamous cell carcinoma, and other pathological subtypes [1,2]. Surgery is still the first choice for early lung cancer. For unresectable locally advanced and metastatic lung cancer other treatment is required. Although there has been much progress in radiotherapy (RT), targeted therapy, immunotherapy, and other treatment methods for unresectable locally advanced or metastatic lung cancer, the median overall survival is still less than two years. The treatment of unresectable locally advanced or metastatic NSCLC depends heavily on RT. Over the past dozen years, RT precision has become increasingly high with the advancement of different radiotherapy modalities, including image-guided radiotherapy (IGRT), intensity-modulated radiotherapy (IMRT), stereotactic radiotherapy (SBRT), etc. [3]. However, the resistance of lung cancer cells to radiation is an urgent problem to be solved in the clinic, especially since the sensitivity of lung adenocarcinoma cells to radiation is less than that of squamous cell carcinoma. Evidence suggests that the existence of cancer stem-like cells (CSCs) or cancer initiation cells is a recognized cause of radio-resistance and chemo-resistance in tumors [4,5,6,7]. From a previous systematic review, we found that carbon ion radiation (C-ions) can effectively kill CSCs depending on their unique physical and biological advantages [8]. The specific mechanism is still being explored.

The stemness of CSCs mainly refers to their self-renewal ability, remodeling, relative silence, metastasis, and treatment resistance [4]. CSCs account for a small proportion of tumor tissue, which may not exceed 0.1–2%. However, they are essential in maintaining tumor growth and regulating tumor metastasis [9,10]. It has been confirmed that CSCs exist in various tumors [11,12,13]. Currently, the sorting of CSCs mainly depends on stem cell surface markers in different cancer cells [5], such as CD133, CD44, and so on [14]. CD44, which has been widely regarded as a CSCs marker in several cancers, has also been shown to have more prominent stemness features in CD44^+^ subpopulations of lung adenocarcinoma [15]. CD44 is dramatically upregulated in surviving radiation cells and could become a marker of radiotherapy response in NSCLC [16]. Additionally, CD44 activation is related to many metabolic pathways, including cell adhesion, proliferation, growth, survival, motility, migration, angiogenesis, and differentiation [17,18]. In summary, it is worth noting that CD44 may be a marker of CSCs in NSCLC.

Autophagy is an intracellular degradation pathway in eukaryotes. Evidence suggests that autophagy affects the radiation sensitivity of tumor cells while maintaining homeostasis by promoting cell survival or causing cell death [19,20]. On one hand, autophagy can be induced by various stimuli, such as redox stress, trophic, energetic stress, and hypoxia. Cell survival is promoted after stress/nutrient restriction by recycling the necessary cellular components [21]. On the other hand, in some cases, autophagy inhibition can inhibit cell death. Due to the extensive crosstalk among different signaling pathways, the pro-death effects of autophagy are very complex. These include autophagy-dependent ferroptosis and autophagic contact necroptosis [22,23]. Studies have shown that autophagy affects the radiation sensitivity of lung adenocarcinoma cells [24,25,26]. However, the role and mechanisms of autophagy in the radiation sensitivity of lung adenocarcinoma CSCs remain unclear.

Transglutaminase-2 (TGM2) is a multifunctional protein primarily found in the cytoplasm and nucleus [27]. TGM2 is associated with multiple biological functions of cells, including apoptosis, autophagy, and cell migration [28,29,30,31]. TGM2 can participate in the regulation of multiple signaling pathways, such as NF-κB signaling pathway, mTOR signaling pathway, etc. [32,33]. In addition, TGM2 is closely associated with tumors and its expression is up-regulated in various cancers. It is related to poor drug response, elevated metastatic potential, and poor patient survival of tumor cells [34,35]. Studies have shown that TGM2 is also associated with the self-renewal and maintenance of stemness characteristics of tumor stem cells, and could become one of the stemness markers of tumor stem cells [36].

Our research found that autophagy and TGM2 contribute to the radio-resistance of CD44^+^A549 cells. CD44 mediates the radio-resistance of CD44^+^A549 stem-like cells through TGM2 regulation of autophagy.

## 2. Materials and Methods

### 2.1. Cell Culture and Cell Sorting

We obtained A549 cells (human NSCLC line) from the American Type Culture Collection. It was cultured in RPMI 1640 medium (with 10% fetal bovine serum (FBS) and 1% antibiotic). CD44^+^A549 cells were obtained by the fluorescence activating cell sorter (FACS) marked with CD44 and cultured in DMEM/F12 (1:1) medium (containing 1xB27 (Gibco, San Francisco, CA, USA), 1xN2 (Gibco, CA, USA), 1% antibiotic (Bioss, Beijing, China), 20 ng/mL bFGF (Peprotech, NJ, USA), 20 ng/mL EGF (Gibco, CA, USA). CD44^+^A549 cells were sorted by FACS from a culture of A549 cells. In short, the A549 cells in logarithmic phase growth were collected and washed in a phosphate-buffered saline (PBS) and incubated with an anti-CD44 antibody (0.2 ug/10^6^ cells) for 30 min. After washing three times, the FITC secondary antibody was added to incubate for 30 min. Then, the fluorescent cells were sorted out by a flow cytometer-BD FACS Aria (Becton Dickinson, Becton Drive Franklin Lakes, NJ, USA).

### 2.2. Ionizing Radiation (IR)

An X-RAD generator (Faxitron, Wheeling, IL, USA) produced the X-rays (2.0 Gy/min, 225 kV, 0.2 mm Al filter). The Heavy Ion Medical Machine (HIMM) at Lanzhou Heavy Ion Hospital provided the carbon ion beam. At the center of the spread-out Bragg peak, there was an ion linear energy transfer of 80 keV/μm, with an energy of 120 MeV/u. The dose rate was 2 Gy/min. A549 and CD44^+^A549 cells were irradiated with 0, 1, 2, 4, 8 Gy X-rays and 0, 1, 2, 4 Gy C-ions, respectively.

### 2.3. Cell Counting Kit-8 (CCK-8) Assay

In 96-well plates, 5 × 10^3^ cells were planted within each well. After 12-h, 24-h, 48-h, and 72-h of irradiation, a CCK-8 reagent (Sunview, Shenzhen, China) was applied to each well for two hours. The optical density (OD) was determined at 450 nm using the microplate reader (Tecan, Männedorf, Switzerland).

### 2.4. EdU Assay

EdU assay kit (C10310-2, RiboBio, Guangzhou, China) was used. The cells were cultured in 6-well plates, supplemented with 50 μmol/L EdU solution in each well, and incubated at 37 °C for 2 h. Subsequently, the cells were treated for 15 min with 4% paraformaldehyde (PFA), 5 min with 2 mg/mL glycine, and 10 min with 0.5% tritonX-100. Then, the cells were incubated with Apollo fluorescent staining solution for 30 min in the dark. After this, they were washed twice with methanol, stained using the 4′, 6-diamidino-2-phenylindole (DAPI) (Vector Laboratories, Burlingame, CA, USA) staining solution at room temperature until 30 min later, and washed with PBS 3 times. The EdU-positive cells were randomly selected and counted under the fluorescence microscope (Olympus Corporation, Tokyo, Japan).

### 2.5. Migration and Invasion

A549 and CD44^+^A549 cells were deposited into 6-well plates. The scratches were obtained using a 10ul pipette tip. After washing with PBS, the cells were grown in a medium devoid of serum. At 0 and 24 h after wounding, the scratches were seen and captured on camera in three randomly selected fields. The wound outcome was obtained using the Image J software, java 1.8.0_172, 64-bit (NIH, Bethesda, MD, USA). The lower side of the transwell chambers (Corning, NY, USA) was covered with Matrigel. In the lower chamber, RPMI 1640 or DMEM/F12 medium with 10% FBS was added, and 50,000 cells were added to the RPMI 1640 or DMEM/F12 medium (serum-free) in the upper chamber. The cells underwent a 24-h culture at 37 °C and 5% CO_2_ before being fixed with 4% PFA and stained using 0.1% crystal violet. Pictures were taken after a cotton swab cleaning of the upper chamber’s inner membrane. The cell counts were determined across three distinct locations using an inverted microscope (Olympus Corporation, Japan).

### 2.6. Measurement of Autophagic Activity and Drugs of Autophagy

The autophagic activity was monitored with the CYTO-ID^®^ Autophagy Detection Kit 2.0 (Enzo Life Sciences, Farmingdale, NY, USA). Cells growing logarithmically were seeded on coverslips, and the medium was carefully removed when the cell density reached 70% confluence level. Washing in assay buffer and incubating cells based on the manufacturer’s instructions followed. After that, the samples were analyzed using a fluorescence microscope (Zeiss LSM-700, Berlin, Germany). The 3-Methyladenine (3-MA) powder (HY-19312, MCE, Monmouth Junction, NJ, USA) was dissolved in cell culture media based on the instructions. The Rapamycin powder (HY-10219, MCE, NJ, USA) was dissolved in dimethyl sulfoxide (DMSO) according to the instructions. Control groups were treated using the same cell culture media/DMSO concentration.

### 2.7. Western Blotting

Protease and phosphorylase inhibitor-containing RIPA Lysis Buffer (Solarbio, Beijing, China) helped generate cell lysates. Using 10%/12% sodium dodecyl sulfate polyacrylamide gel electrophoresis (SDS-PAGE), the samples were electrophoresed and then transferred to 0.22 µm or 0.45 µm polyvinylidene fluoride (PVDF) membranes (Millipore, St. Louis, MA, USA). Then, the secondary antibody was incubated on the membrane after the primary antibody incubation. An enhanced chemiluminescence (ECL) chromogenic substrate (NCM Biotech, Suzhou, China) was used for color development. Table S1 contains the required antibodies.

### 2.8. Nontargeted LC–MS-Based Metabolomics

The irradiated cell culture medium (X-rays-4 Gy-48 h, C-ions-2 Gy-12 h) from the two cells was collected. Each sample had four parallel samples. The acquired MS data were performed using the XCMS software (https://xcmsonline.scripps.edu, accessed on 15 August 2023). The online KEGG and HMDB databases helped annotate the metabolites by matching the exact molecular mass data (*m*/*z*) of the samples with the database. Student’s *t*-tests were used to find variations in metabolite concentrations between the two phenotypes. MetaX, principal component analysis (PCA), and partial least squares data analysis (PLS-DA) were used to help distinguish the various variables between groups. The changed metabolites between the two groups were determined using statistical analysis (*p* < 0.05) and variable importance in the projection (VIP) > 1 from PLS-DA. Through using metabolomics pathway analysis (http://www.metaboanalyst.ca/, accessed on 20 August 2023), the changed metabolites and the connection of possible pathways were examined. Utilizing the KEGG database (http://www.kegg.jp/, accessed on 20 August 2023), metabolite roles in the metabolic pathways were deciphered.

### 2.9. RNA Interference

GenePharma (Shanghai, China) provided the small interfering RNAs (siRNAs). Cells were cultured in 6-well plates. Then, we transfected cells with siRNAs using GP-transfect-Mate (G04009, GenePharma). All the experiments were performed at 48 h post-transfection. The RNA interference sequences are listed in Table S2.

### 2.10. RT-qPCR and PCR Array

As directed by the manufacturer, total RNA was extracted using the TRIzol reagent (Invitrogen, Carlsbad, CA, USA). The EasyScript One-Step gDNA Removal and cDNA Synthesis SuperMix (TRANSGEN, Beijing, China) was used to reverse transcribe each RNA sample into complementary DNA (cDNA). QuantStudio^TM^ 5 Systems (Thermofisher Scientific, MA, USA) were used for RT-qPCR. Using GAPDH as a housekeeping gene, the conventional 2^−ΔΔCt^ method helped calculate the relative RNA abundances. A Human Autophagy PCR Array (Wcgene Biotech, Shanghai, China) helped evaluate gene expression profiles.

### 2.11. Immunofluorescence (IF)

Cells were fixed for 15 min at room temperature. Then, PFA was removed and washed thrice using PBS for 5 min. Next, cells were incubated for 20 min for cell permeabilization with 0.5% Triton X-100 liquid and washed three times as in the previous step. The samples were then placed in 5% albumin from bovine serum (BSA) and incubated for 2 h at room temperature. Then, the primary antibody was added and incubated overnight. The next day, after removing the primary antibody, the secondary antibody was kept in the dark for 1 h. Finally, an anti-fluorescence attenuating mountant containing DAPI was added to capture images with a fluorescence microscope. Table S1 lists the needed antibodies. The cells’ pictures were captured with a confocal microscope.

### 2.12. Bioinformatics Analysis

An online website (https://www.xiantaozi.com/, accessed on 7 October 2023) was used to analyze the molecular expression correlation of CD44, TGM2, and LC3B, and they were plotted with Origin Ver. 2023 (OriginLab Corporation, Northampton, MA, USA).

### 2.13. Co-Immunoprecipitation (Co-IP)

The cell lysates were obtained according to the instructions (ACE Biotechnology, Nanjing, China). The steps to rinse rProtein A/G MagPoly Beads were followed, then 2 μg anti-LC3B antibody, or anti-IgG antibodies were added with rProtein A/G MagPoly Beads and incubated for 7 h at 4 °C. Then, 500 ug of protein lysates were added and incubated at 4 °C overnight. The rProtein A/G MagPoly Beads were washed three times. The protein samples were added to 50 μL of 1 × loading buffer, the sample was placed in a metal bath and heated at 100 °C for 10 min to undergo Western blotting.

### 2.14. Animal Experiments

The Ethical Review of Experimental Animals of the Institute of Modern Physics, Chinese Academy of Sciences approved all animal experiments of this study (Approval No. 2024-015). A549 cells and CD44^+^A549 cells (5 × 10^6^/100uL PBS) were inoculated subcutaneously into fossa axillaries of 5-week-old male nude BALB/c mice (GemPharmatech Co., Ltd., Nanjing, China) (*n* = 4 per group). The mice were housed in a facility that was specifically designed to eliminate pathogens, with a temperature of 23 ± 2 °C, 50–60% humidity, a light/dark cycle of 12/12 h, free water access, and a regular rodent chow diet. The tumor weight, length, and width was measured every four days until 28 days after injection; tumor volume (mm^3^) = (length × width^2^)/2.

### 2.15. Statistical Analysis

Statistical analysis and production of graphs were performed using SPSS software v27.0 (Chicago, IL, USA) and GraphPad Prism software V9.1.1 (GraphPad Software, Inc., San Diego, CA, USA). Quantitative results are represented as means ± standard deviations (SD). Comparisons between treatment groups were made by unpaired two-tailed Student’s *t*-test or two-way ANOVA with Tukey’s multiple comparison test, where appropriate. A significant difference was defined as *p* < 0.05.

## 3. Results

### 3.1. Identification of “Stemness” of CD44^+^A549 Cells

**Figure 1:**
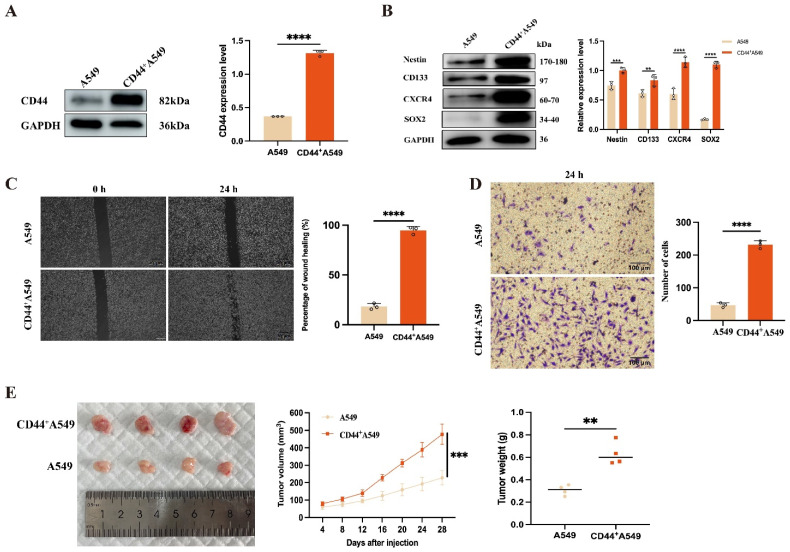
Identification of “stemness” of CD44^+^A549 cells. (**A**) Western blot identified CD44 expression in cells sorted by FACS; (**B**) the expression of stemness factors in CD44^+^A549 cells; (**C**) comparison of the migration capacity of A549 and CD44^+^A549 cells by wound healing experiments after 24 h; (**D**) comparison of the invasion capacity of A549 and CD44^+^A549 cells by transwell after 24 h; (**E**) comparison of tumorigenicity of A549 and CD44^+^A549 cells in vivo (*n* = 4 per group). The weight, length, and width of the tumors were measured every 4 days until 28 days after injection, tumor volume (mm^3^) = (length × width^2^)/2. ** *p* < 0.01, *** *p* < 0.001, **** *p* < 0.0001.

CD44^+^A549 cells were sorted from A549 cells using the FACS technology, with a positive rate of 99.5 ± 0.1% (Figure S1). At the protein level, it was clear that compared with A549 cells, the expression of CD44 in the sorted cells was significantly increased (Figure 1A), hereafter called CD44^+^A549 cells. Subsequently, we analyzed whether CD44^+^A549 cells had “stemness” characteristics by stemness factors (CD133, SOX2, Nestin, CXCR4), wound healing, and transwell experiments. Under similar conditions, the stemness factors, the ability of migration, and invasion of CD44^+^A549 cells were significantly higher than those of A549 cells, *p* < 0.0001 (Figure 1B–D). As the gold standard for verifying “stemness,” we observed the tumorigenicity of both cells in animal experiments. After 28 days of tumor cell transplantation, CD44^+^A549 cell tumorigenicity was significantly improved (Figure 1E).

### 3.2. CD44^+^A549 Cells Exhibit Greater Radio-Resistance than A549 Cells, and C-Ions Could Overcome This Radio-Resistance

**Figure 2:**
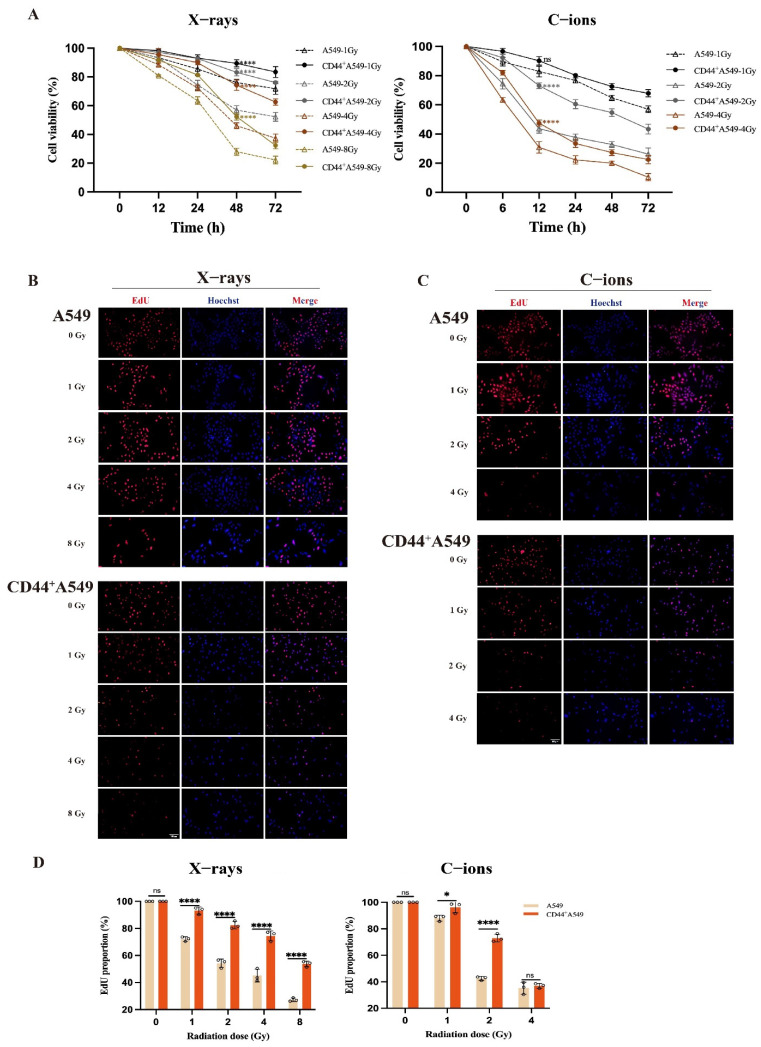
CD44^+^A549 cells have more obvious radio-resistance than A549 cells, and C-ions could overcome this radio-resistance. (**A**) Changes in cell viability of A549 and CD44^+^A549 cells at different time points and doses after X-rays (**left**) and C-ions (**right**) irradiation by CCK-8 assay; (**B**) changes in cell viability of A549 and CD44^+^A549 cells at 48 h after irradiation of X-rays by EdU assay; (**C**) changes in cell viability of A549 and CD44^+^A549 cells at 12 h after irradiation of C-ions by EdU assay; (**D**) statistical analysis of (**B**,**C**); ns: no significance, * *p* < 0.05, **** *p* < 0.0001. Magnifying objective: 20×.

The two cells were irradiated with different doses of two types of radiation (X-rays and C-ions), then the OD values at 450 nm (Figure 2A) were measured and processed. The proliferation viability of CD44^+^A549 cells was significantly higher than that of the A549 cells after 48 h of 4 Gy X-rays and 12 h of 2 Gy C-ions irradiation (the mean values ± SD are: 74.24 ± 3.610% vs. 46.07 ± 2.094%, 73.05 ± 1.954% vs. 43.65 ± 3.103%, *p* < 0.0001, respectively) (Figure S2). Based on this result, X-rays 4 Gy-48 h and C-ions 2 Gy-12 h were selected as doses and time points for experimental follow-up studies. Summarizing the results of CCK-8, the viability of CD44^+^A549 cells is higher than that of A549 cells. In addition, we performed EdU experiments on these two cells under different doses of irradiation and the results were the same as CCK-8 (Figure 2B–D). We found that under the condition of 2 Gy C-ions, the killing effect on cells is more potent than that of 4 Gy X-rays.

Overall, CD44^+^A549 cells have more obvious radio-resistance than A549 cells. Moreover, the killing effect of 2 Gy C-ions on CD44^+^A549 cells is more severe than that of 4 Gy X-rays, and it is gratifying that C-ions can overcome radio-resistance to a certain extent.

### 3.3. CD44^+^A549 Cells Possess High Levels of Autophagy, CD44^+^A549 Cells Regulates Its Radiation Sensitivity through Autophagy

**Figure 3:**
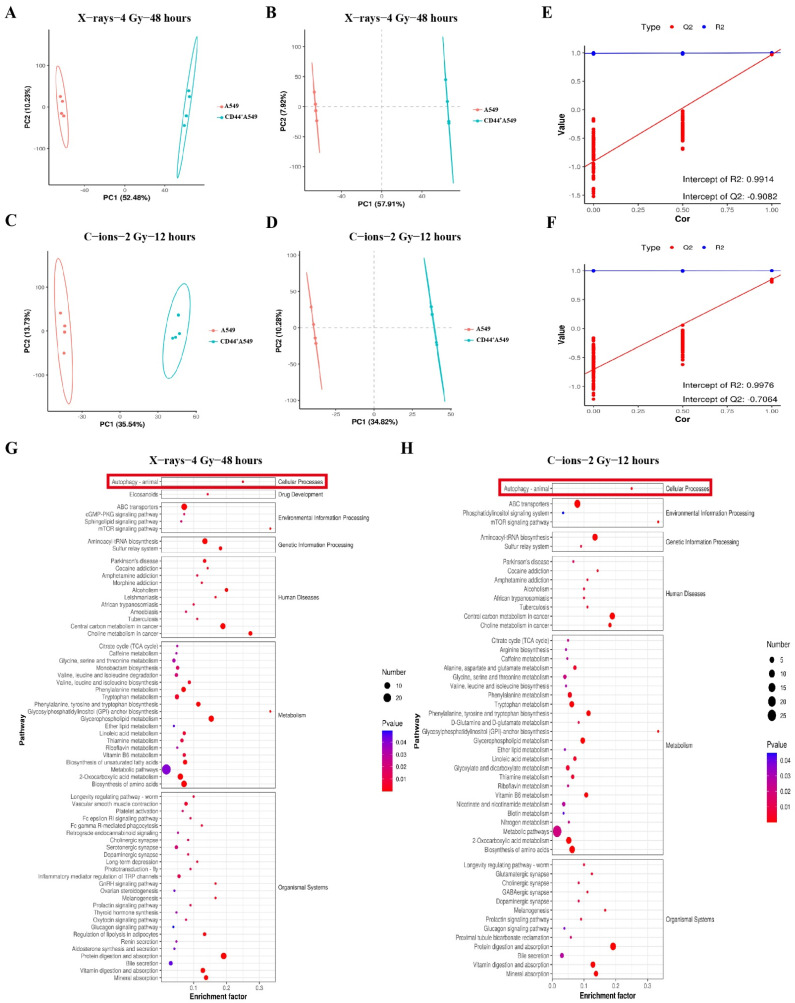
LC–MS showed significant differences in autophagy levels between A549 cells and CD44^+^A549 cells. (**A**,**C**) PCA of the A549 cells and CD44^+^A549 cells irradiated by X-rays and C-ions; (**B**,**D**–**F**) PLS-DA plot of the A549 and CD44^+^A549 cells irradiated by X-rays and C-ions; (**E**,**F**) validation of the PLS-DA model by the 200-time permutation test; (**G**,**H**) KEGG analysis of these metabolites in various metabolic pathways, X-rays (**left**), C-ions (**right**). The red box shows the level of enrichment in autophagy.

A supervised multivariate statistical analysis was performed using PLS-DA to screen differential metabolites. A significant separation between the A549 and CD44^+^A549 groups was observed in the PCA and PLS-DA (Figure 3A–D). The R2 and Q2 values of different groups from the 200 permutation tests demonstrated that the PLS-DA model was reliable without overfitting (Figure 3E,F). The enrichment analysis of the KEGG pathways showed that the differential metabolites were enriched in the metabolic pathways (Figure 3G,H). We can observe that autophagy depicts a significant difference between the two cell lines, both after C-ions radiation and after X-rays radiation. Combined with previous reports on the role of autophagy in cellular radiation sensitivity, it is speculated whether autophagy plays a role in the radiation resistance of CD44^+^A549 cells here.

**Figure 4:**
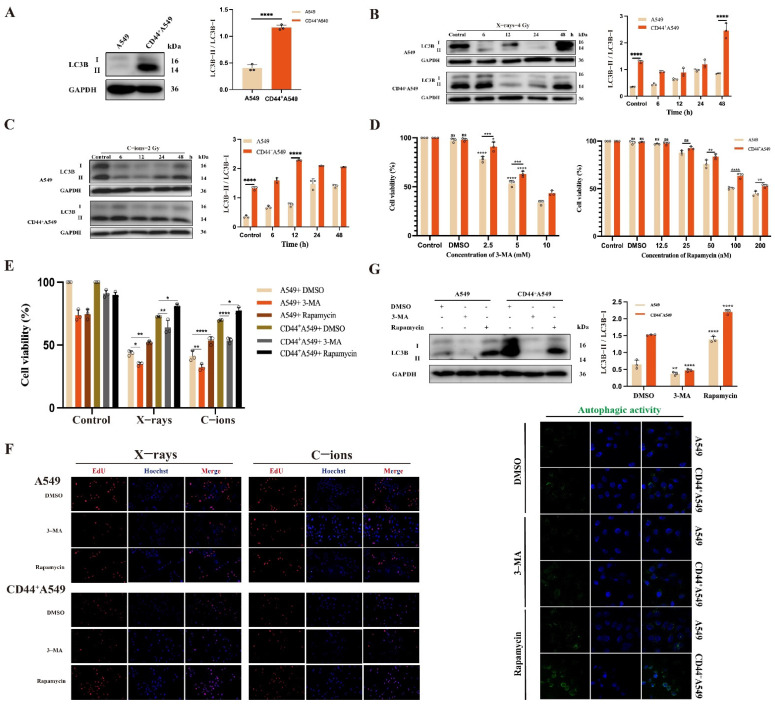
CD44^+^A549 cells regulate their radiation sensitivity by autophagy. (**A**) Background expression levels of autophagy (LC3B-II/LC3B-1) in A549 and CD44^+^A549 cells and statistical analysis; (**B**) changes of autophagy levels of A549 and CD44^+^A549 cells at different time points after exposure to 4 Gy X-rays and statistical analysis; (**C**) changes of autophagy levels of A549 and CD44^+^A549 cells at different time points after exposure to 2 Gy C-ions and statistical analysis; (**D**) concentration screening of 3-MA and Rapamycin; (**E**,**F**) changes in cell viability of A549 and CD44^+^A549 cells after 48 h of drug administration (3-MA and Rapamycin) by CCK-8 and EdU assay, magnifying objective: 20×. (**G**) Changes in autophagy levels in A549 and CD44^+^A549 cells after 48 h of drug administration (3-MA and Rapamycin), Western blot (**above**) and measurement of autophagic activity (**bottom**), magnifying objective: 40× oil lens. ns: no significance, * *p* < 0.05, ** *p* < 0.01, *** *p* < 0.001, **** *p* < 0.0001.

In Figure 4A, the autophagy level (shown as LC3B-II/LC3B-I) was increased in CD44^+^A549 cells than in A549 cells (*p* < 0.0001). Subsequently, we analyzed the relationship between autophagy and cell proliferation activity after irradiation, as shown in Figure 4B,C. The data indicates increased autophagy levels of A549 and CD44^+^A549 cells after irradiation. Moreover, the autophagy levels in the two cell types have the most statistical differences at X-rays 4 Gy-48 h and C-ions 2 Gy-12 h, consistent with the results of cell proliferation activity. It indicates a possible correlation between autophagy and cell proliferation activity. To verify this hypothesis, CCK-8 was used to screen the effects of different drug concentrations (Rapamycin and 3-MA) on cell viability after 48 h, and finally selected the following concentrations (Rapamycin, 50 nM and 3-MA, 2.5 mM) (Figure 4D). An autophagy activator (Rapamycin) or autophagy inhibitor (3-MA) was used to explore the impact of autophagy on cell proliferation using the CCK-8 and EdU assays (Figure 4E,F). Compared with the control group, the proliferation activity of the two cell lines was significantly decreased in the 3-MA group alone, particularly in the C-ions group. In contrast, after the cells were exposed to radiation, the autophagy activation group could significantly increase the proliferation activity of the irradiated cells compared to the DMSO group. Meanwhile, the effectiveness of 3-MA and Rapamycin on autophagy was verified at the protein level and the autophagic activity, respectively, in Figure 4G. The results suggested that autophagy can protect CD44^+^A549 and A549 cells from ionizing radiation by increasing proliferation activity.

### 3.4. TGM2 Is Up-Regulated in CD44^+^A549 Cells, CD44^+^A549 Cell Regulates Radiation Sensitivity by TGM2

**Figure 5:**
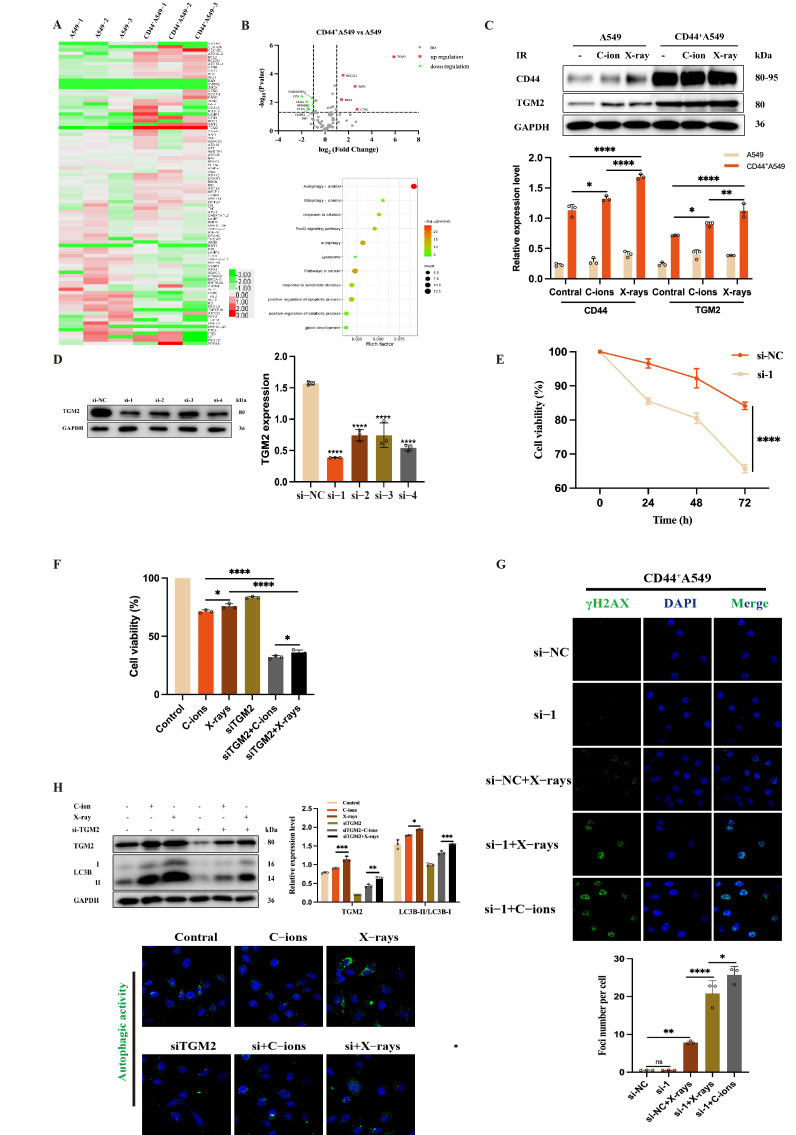
TGM2 is up-regulated in CD44^+^A549 cells and CD44^+^A549 cells regulate radiation sensitivity by TGM2. (**A**) Expression heat map analysis of 91 genes related to autophagy in A549 and CD44^+^A549 cells, each with three separate samples by PCR assay; (**B**) volcanic map of genes up-regulated and down-regulated in CD44^+^A549 cells compared to A549 in 91 genes (above), and enrichment analysis of differentially expressed genes (bottom); (**C**) changes of CD44 and TGM2 in A549 and CD44^+^A549 cells irradiated by 4 Gy X-rays and 2 Gy C-ions; (**D**) efficiency of small interfering RNAs silencing TGM2 of CD44^+^A549 cells; (**E**) the cell viability after siTGM2 of CD44^+^A549 cells by CCK-8 assay; (**F**) the cell viability after siTGM2 of CD44^+^A549 cells irradiated by X-rays and C-ions; (**G**) effect of radiation combined with siTGM2 on DNA DSBs in CD44^+^A549 cells, magnifying objective: 40× oil lens; (**H**) changes of TGM2 and LC3B after siTGM2 of CD44^+^A549 cells irradiated by X-rays and C-ions (above), and measurement of autophagic activity (bottom), magnifying objective: 40× oil lens; ns: no significance, * *p* < 0.05, ** *p* < 0.01, *** *p* < 0.001, **** *p* < 0.0001.

**Figure 6:**
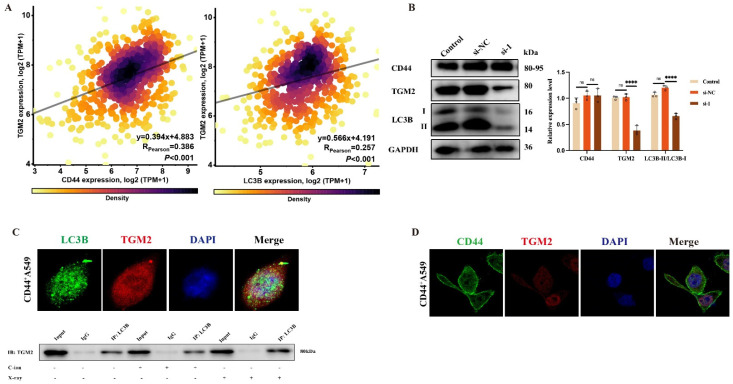
Relationship between TGM2, CD44 and LC3B. (**A**) bioinformatic analysis was used to analyze the correlation between CD44, TGM2, and LC3B expressions; (**B**) Western blot identified the expression of CD44, TGM2, and LC3B after siTGM2 of CD44^+^A549 cells; (**C**) immunofluorescence colocalization showed that TGM2 and LC3B had a colocalization effect (above) in CD44^+^A549 cells, Co-IP analysis of molecular (TGM2 and LC3B) interactions (bottom), magnifying objective: 40× oil lens. (**D**) Immunofluorescence colocalization showed that TGM2 and CD44 had no colocalization effect, magnifying objective: 40× oil lens. ns: no significance, **** *p* < 0.0001.

The mRNA expression of 91 autophagy-related genes in a PCR array was used to identify the differentially expressed genes in two cell types (Figure 5A). Table 1 indicates that among the 91 autophagy-related genes, 11 genes of CD44^+^A549 cells were up-regulated, and 10 genes were down-regulated compared with A549 cells. From the volcanic map of Figure 5B, mRNA of TGM2 expression was the most up-regulated (*p* < 0.001). Subsequently, a bioinformatics analysis was used to analyze TGM2. The results showed that there was a certain correlation between TGM2, CD44, and LC3B in lung adenocarcinoma cells (Figure 6A). Figure 5C shows that the protein expression of TGM2 increased in the two cell lines exposing 2 Gy C-ions and 4 Gy X-ray radiation. The data above indicated that TGM2 is associated with radio-resistance.

To explore the role of TGM2, CD44^+^A549 cells were transfected with siRNAs to knock down TGM2 expression. Among the four siRNAs, only si-1 possessed a knockout efficiency greater than 70% as shown in Figure 5D. The cell viability of CD44^+^A549 after siTGM2 was significantly lower than that of the control group (Figure 5E). Subsequently, TGM2^−^CD44^+^A549 cells were irradiated by IR at 2 Gy C-ions and 4 Gy X-rays. The proliferation activity of CD44^+^A549 cells is further inhibited by treatment with si-1 and IR (Figure 5F). Interestingly, through the IF of gamma H2AX, it was found that the combined IR groups have more gamma H2AX, indicating that siTGM2 can increase the radiation sensitivity of CD44^+^A549 to IR, resulting in more DNA DSBs. This phenomenon was more prominent in the combined C-ions group: 2 Gy C-ions cause more gamma H2AX to CD44^+^A549 cells than 4 Gy X-rays (Figure 5G). In Figure 5H, the autophagy level of TGM2^−^CD44^+^A549 cells significantly decreased compared with the control, with increasing TGM2 and LC3B protein expressions after IR. In summary, these results demonstrate that high expression of TGM2 leads to the radio-resistance of CD44^+^A549 cells, and it may be associated with autophagy.

### 3.5. TGM2 Regulates Autophagy by Interacting with LC3B within CD44^+^A549 Cells

The decline of protein expression of LC3B in TGM2^−^CD44^+^A549 cells indicated that LC3B could be a downstream molecule of TGM2. As presented in Figure 6A, bioinformatics analysis reveals no change in CD44 protein expression within TGM2^−^CD44^+^A549 cells. However, A549 cells, with low CD44 expression, have lower TGM2 levels. It has also been validated at the protein level (Figure 6B). These results indicated that CD44 could regulate TGM2 expression, so interactions between TGM2, LC3B, and CD44 were measured using IF. The co-localization between TGM2 and LC3B in CD44^+^A549 cells is presented in Figure 6C. IF and Co-IP experiments could prove the interaction between TGM2 and LC3B in CD44^+^A549 cells. Unfortunately, no direct interaction could be observed between CD44 and TGM2 (Figure 6D). So far, we believe TGM2 regulates autophagy by interacting with LC3B in CD44^+^A549 cells and establishing the CD44-TGM2-LC3B regulatory axis in CD44^+^A549 cells to regulate cell radiation sensitivity.

## 4. Discussion

In this study, CD44^+^A549 cells with radio-resistance and stemness were used as the main research object. The results established that radiation-induced autophagy plays a pivotal role in the radio-resistance of CD44^+^A549 cells. After high-throughput sequencing using PCR array, TGM2 was obtained as our main target molecule, and its role in the radio-resistance of CD44^+^A549 cells was verified using siRNA. Together, IF and Co-IP proved that TGM2 can interact with LC3B to regulate the autophagy level in CD44^+^A549 cells, and CD44 can affect TGM2 expression upstream. In addition, we found that 2 Gy C-ions had a more significant killing effect on CD44^+^A549 cells than 4 Gy X-rays.

We identified a population of CD44^+^A549 stem-like cells using FACS, accounting for 99.5 ± 0.1%. It was determined that they had a stronger proliferation, migration, invasion, tumorigenicity, and radio-resistance ability than the A549 cells. CD44 is a non-kinase cell surface transmembrane glycoprotein with a pivotal role in controlling the tumorigenic features of tumor cells. This could lead to tumor development, metastasis, and resistance to chemotherapy [37,38,39]. In our study, CD44^+^A549 cells and A549 cells showed great differences in proliferation, migration, invasion, and radiation sensitivity to IR (X-rays and C-ions), indicating that the high expression of CD44 molecules is critical in the characteristics of CD44^+^A549 cells. Follow-up studies have demonstrated that the CD44 expression did not decrease after TGM2 knockdown in CD44^+^A549 cells with siRNA. Therefore, CD44 is required upstream of the TGM2 molecule to regulate autophagy, but IF colocalization experiments showed no direct interaction between the two molecules.

CSCs are typically characterized by high autophagy levels and play various roles: (1) maintaining cellular pluripotency; (2) coping with low nutrient and hypoxia levels in the tumor microenvironment; (3) regulating CSCs migration and invasion; (4) promoting resistance to chemoradiotherapy; and (5) helping evade immune surveillance. High autophagy levels have also been confirmed in our experiments. On one hand, autophagy plays a protective role in cells. On the other hand, it causes autophagic cell death. In our study, we found that autophagy was induced to increase after the irradiation of cells. The autophagy level of the CD44^+^A549 cells was significantly higher than that of the A549 cells at X-rays 4 Gy-48 h and C-ions 2 Gy-12 h. Moreover, the proliferation activity of CD44^+^A549 cells was also significantly higher than that of A549 cells at the same time, indicating that radiation-induced autophagy has a protective effect on cells.

3-MA is an autophagy inhibitor and Rapamycin is an autophagy activator. The combination group of IR and 3-MA could further inhibit cell proliferation than the IR alone group, and IR combined with Rapamycin could reduce the damaging effect of IR on cells. Autophagy plays a critical role in regulating radiation sensitivity, and its inhibition can enhance the radiation sensitivity of cells, consistent with previous studies [20,40,41,42]. Based on this, targeted autophagy could be an effective means to study tumor sensitization. This is especially important when combining RT with drugs for autophagy because radiation-induced autophagy can either promote death or survival depending on the situation [43]. The dependence of autophagy on radiation dose and time, and its effects on cells also need to be explored by more research.

Next, TGM2 was screened by identifying differences in autophagy-related gene expression between the two cells. Zhang et al. observed that inhibiting the TGM2 expression decreases cell proliferation and TGM2-induced chemo-resistance [44]. This study also observed that TGM2 reactivity increased after exposure to radiation in the two cells. Thus, TGM2 may be a correlated factor impacting radiation resistance. We transfected CD44^+^A549 cells with siRNA to silence TGM2 expression and significantly reduced cell viability. The cell viability decreased further after the cells were irradiated. All in all, TGM2 contributes to the radiation resistance of CD44^+^A549 cells. Coincidentally, previous studies have proved that TGM2 was upregulated in Glioblastoma patients and associated with tumor progression and poor prognosis [45,46].

Bioinformatics analysis showed that there was a certain correlation between the expression of CD44 and TGM2, TGM2, and LC3B. The results also proved the following: the expressions of CD44, TGM2, and LC3B were significantly increased in CD44^+^A549 cells than in A549 cells. Most importantly, Co-IP revealed that TGM2 could interact with LC3B to regulate autophagy. In this regard, Zheng et al. determined that the TGM2 contained two potential LC3-interacting region (LIR) motifs [47], explaining the interaction between the two molecules. It is generally accepted that many molecules can directly activate autophagy by binding to LC3 via LIR, such as OPTN, NBR1, FUNDC1, etc.

From another point of view, C-ions (2 Gy) could suppress the viability of CD44^+^A549 cells more strongly than X-rays (4 Gy). Figure 4C shows that the CD44 and TGM2 expression levels were increased to varying degrees after irradiation. Moreover, the X-ray (4 Gy) group was significantly higher than the control group. We have previously shown that CD44 and TGM2 are associated with the radiation resistance of CD44^+^A549 cells, indicating that C-ions can inhibit the radiation resistance of related molecules compared to X-rays. After siTGM2, the combined C-ions (2 Gy) induced more DNA DSBs than X-ray (4 Gy), which has been similarly confirmed. This is a comparison of the physical doses of the two rays. Still, for the following two reasons, the relative biology effectiveness value of CD44^+^A549 cell for C-ions could not be obtained, so no biological dose comparison could be made. We performed clone formation experiments of CD44^+^A549 cells at the beginning. However, the results were not available due to the following reasons: 1. The clones formed by CD44^+^A549 cells are sand-like scattered and do not agglomerate into a single clone unit, affecting the final counting; 2. The attachment of CD44^+^A549 cells is not tight, and the cells floating in the culture medium are also viable, resulting in an inaccurate final survival score. Together, C-ions (2 Gy) could inhibit the viability of CSCs of NSCLC than X-ray (4 Gy).

We demonstrated that inhibition of autophagy and TGM2 could improve the radiation sensitivity of CSCs in vitro. However, more in-depth explorations are required regarding regulatory mechanisms, including the role of inhibition of autophagy and TGM2 on tumors in vivo. This is also the key experimental direction of our subsequent work. Future research should focus on translating basic research results in vitro better to benefit tumor treatment in vivo and clinical cancer.

**Figure 7:**
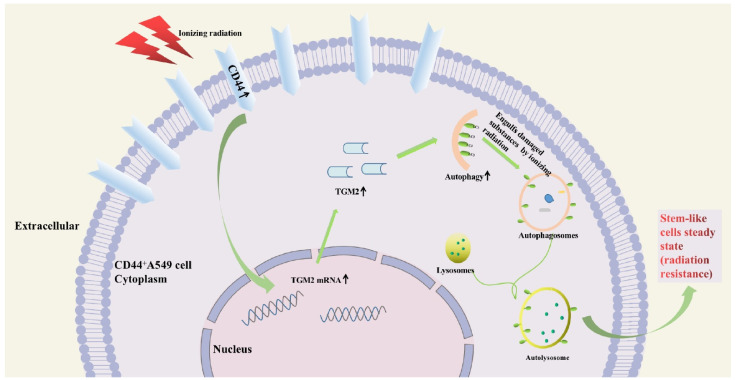
Diagram of the main mechanism of this study. CD44^+^A549 stem-like cells possess high TGM2 and autophagy levels. When CD44^+^A549 stem-like cells are exposed to IR, TGM2 expression is further upregulated, which can interact with the autophagy hallmark molecule LC3B to upregulate the level of autophagy. Autophagy, which relies on the action of lysosomes, can maintain a steady state inside and outside cells by phagocytosis and lysis of organelles damaged by external stimuli. TGM2-mediated upregulation of autophagy enhances resistance to external injury stimuli of stem-like cells, which may be one of the important reasons for the radiation resistance of NSCLC stem-like cells with positive CD44 expression.

In summary (Figure 7), TGM2 and autophagy are associated with radio-resistance of CD44^+^A549 cells. TGM2 depletion and autophagy inhibition can strengthen the radiation sensitivity of CD44^+^A549 cells to X-rays and C-ions. In addition, C-ions (2 Gy) have a more substantial killing effect on CD44^+^A549 cells than X-rays (4 Gy). Further studies revealed that TGM2 can interact with LC3B to regulate autophagy, facilitating the radio-resistance of CD44^+^A549 cells. Our study may provide new biomarkers and strategies to alleviate the radio-resistance of CSCs of NSCLC.

## Figures and Tables

**Table 1:** Statistically different genes in fold change (FC) and *p* values for CD44^+^A549 and A549 cells.

Gene Name	CD44^+^A549/A549 FC	*p* Value	Difference Labeling
AKT1	1.916798987	0.031498127	*
ARSB	0.540021603	0.007457067	**
ATG4B	1.178883886	0.024688472	*
ATG7	1.606276736	0.019764521	*
ATG9A	1.563050332	0.002539375	**
BCL2	2.661168151	0.006385583	**
BCL2L1	2.900310928	0.000126638	***
BECN1	1.411060457	0.023430197	*
CLN3	0.568644846	0.042009251	*
CTSS	6.700339499	0.031175413	*
DAPK1	0.386402211	0.028667488	*
GABARAPL1	0.468758375	0.004627373	**
HGS	1.76961699	0.000679948	***
PIK3R4	0.589085589	0.007750301	**
PTEN	0.353429337	0.025497525	*
RB1	0.485138608	0.044292159	*
RPS6KB1	0.47761233	0.013796305	*
TGM2	59.24354241	6.42 × 10^−6^	***
TP53	0.251151782	0.003567178	**
ULK2	0.334679544	0.008775852	**
WIPI1	5.937977239	0.000775029	***

Note: * *p* < 0.05, ** *p* < 0.01, *** *p* < 0.001.

## Data Availability

Research data are stored in an institutional repository and will be shared upon reasonable request to the corresponding authors.

## Supplementary Materials

The following supporting information can be downloaded at: https://www.mdpi.com/article/10.3390/biomedicines12102231/s1, Table S1: Antibodies used in the experiments; Table S2: Target sequences of siRNAs. Figure S1: Flow cytometry diagram of sorted cells by FACS; Figure S2: The specific values extracted from Figure 2.
