# Supplementary material for: TGM2-Mediated Autophagy Contributes to the Radio-Resistance of Non-Small Cell Lung Cancer Stem-like Cells
Source: Biomedicines. 2024 Sep 30;12(10):2231. doi: 10.3390/biomedicines12102231 (PMC11504678; doi:10.3390/biomedicines12102231)
Supplement: Supplementary file 1 [file biomedicines-12-02231-s001.zip › Supplementary tables.pdf]

Table S1. Antibodies used in the experiments.

Antibody	Supplier	Catalog #	Application
CD44	Proteintech	15675-1-AP	WB, IB (1:5000),<br>IF (1:200)
LC3B	Cell Signaling Technology	43566S	WB (1:1000),<br>IF (1:200),<br>IP (2μg)
TGM2	Proteintech	15100-1-AP	WB, IB (1:5000),<br>IF (1:200)
CXCR4	Proteintech	60042-1-Ig	WB (1:1000-1:4000)
CD133	Proteintech	66666-1-Ig	WB (1:2000-1:10000)
Nestin	Proteintech	19483-1-AP	WB (1:500-1:1000)
SOX2	Proteintech	11064-1-AP	WB (1:500-1:1000)
IgG	Proteintech	30000-0-AP	IP (2μg)
HRP-conjugated Affinipure Goat Anti-Mouse IgG(H+L)	Proteintech	SA00001-1	WB (1:5000)
HRP-conjugated Affinipure Goat Anti-Rabbit IgG(H+L)	Proteintech	SA00001-2	WB (1:5000)
CoraLite488-conjugated Goat Anti-Mouse IgG(H+L)	Proteintech	SA00013-1	IF (1:500)
CoraLite488-conjugated Goat Anti-Rabbit IgG(H+L)	Proteintech	SA00013-2	IF (1:500)
CoraLite594 – conjugated Goat Anti-Mouse IgG(H+L)	Proteintech	SA00013-3	IF (1:250)
CoraLite594 – conjugated Goat Anti-Rabbit IgG(H+L)	Proteintech	SA00013-4	IF (1:250)
GAPDH	Proteintech	60004-1-Ig	WB (1:100000)

Note: WB: Western blot, IB: Immunoblotting, IF: Immunofluorescence.

Table S2. Target sequences of siRNAs.

Genes	Forward primer (5′-3′)	Reverse primer (5′-3′)
siTGM2-1	GAGGAAGUUAAGGUGAGAATT	UUCUCACCUUAACUUCCUCTT
siTGM2-2	GGCUGAAGAUCAGCACUAATT	UUAGUGCUGAUCUUCAGCCTT
siTGM2-3	GCCUGAUCCUUCUAGAUGUTT	ACAUCUAGAAGGAUCAGGCTT
siTGM2-4	GUCGUGACCAACUACAACUTT	AGUUGUAGUUGGUCACGACTT
GAPDH	GGCACCCAGCACAATGAAG	CCGATCCACACGGAGTACTTG

Note: siRNAs: Small interfering RNAs.
