# Supplementary figures and images for: TGM2-Mediated Autophagy Contributes to the Radio-Resistance of Non-Small Cell Lung Cancer Stem-like Cells
Source: Biomedicines. 2024 Sep 30;12(10):2231. doi: 10.3390/biomedicines12102231 (PMC11504678; doi:10.3390/biomedicines12102231)

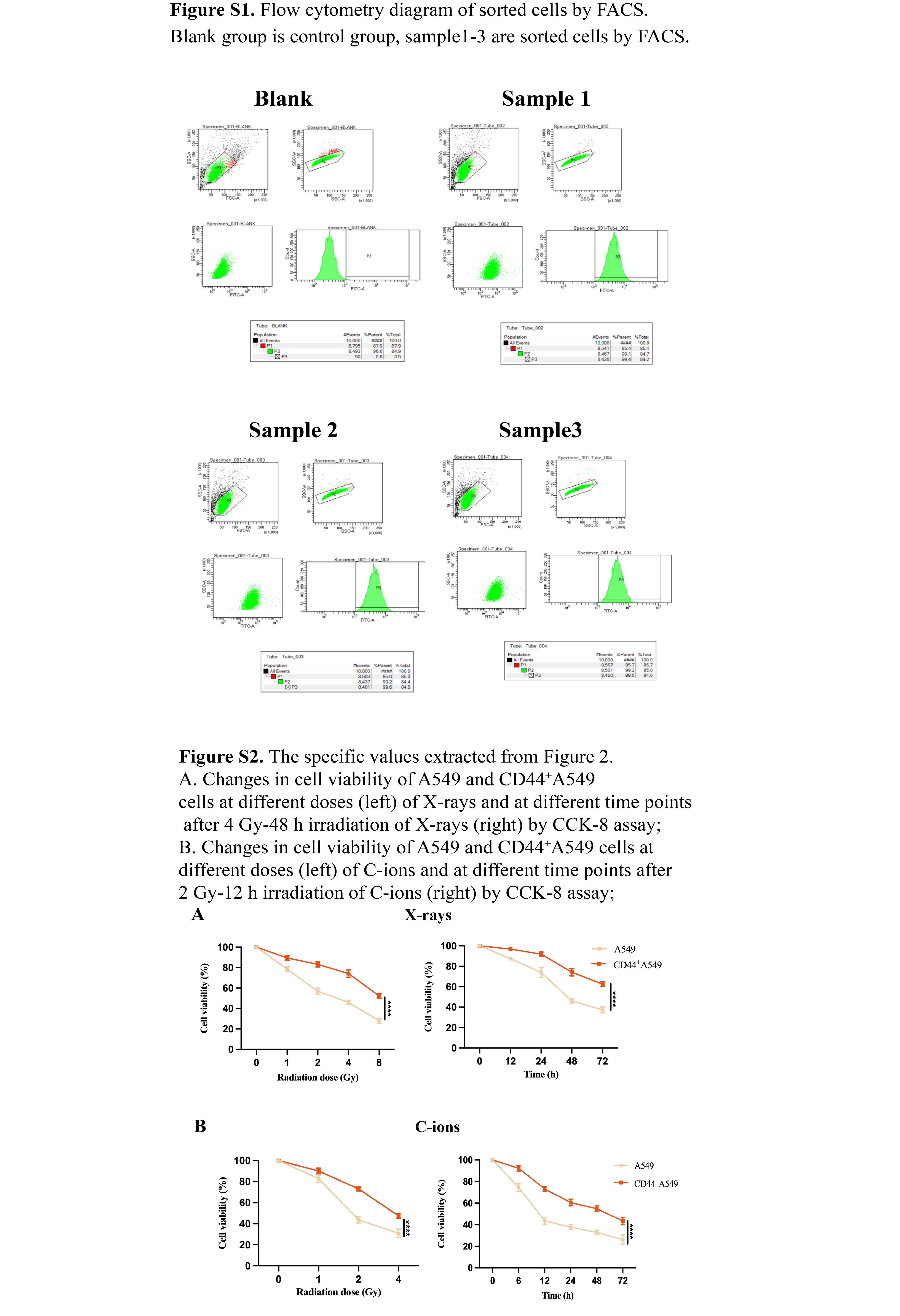

Supplement: Supplementary file 1 [file biomedicines-12-02231-s001.zip › Supplementary figures.jpg]
